# Brain laterality evaluated by F-18 fluorodeoxyglucose positron emission computed tomography in autism spectrum disorders

**DOI:** 10.3389/fnmol.2022.901016

**Published:** 2022-08-10

**Authors:** Keattichai Keeratitanont, Daris Theerakulpisut, Narong Auvichayapat, Chanyut Suphakunpinyo, Niramol Patjanasoontorn, Somsak Tiamkao, Supatporn Tepmongkol, Benjapa Khiewvan, Yutapong Raruenrom, Piyawan Srisuruk, Suchat Paholpak, Paradee Auvichayapat

**Affiliations:** ^1^Division of Nuclear Medicine, Department of Radiology, Faculty of Medicine, Khon Kaen University, Khon Kaen, Thailand; ^2^Noninvasive Brain Stimulation Research Group of Thailand, Faculty of Medicine, Khon Kaen University, Khon Kaen, Thailand; ^3^Department of Pediatrics, Faculty of Medicine, Khon Kaen University, Khon Kaen, Thailand; ^4^Department of Psychiatry, Faculty of Medicine, Khon Kaen University, Khon Kaen, Thailand; ^5^Department of Medicine, Faculty of Medicine, Khon Kaen University, Khon Kaen, Thailand; ^6^Division of Nuclear Medicine, Department of Radiology, Faculty of Medicine, Chulalongkorn University, Bangkok, Thailand; ^7^Chulalongkorn University Biomedical Imaging Group (CUBIG), Faculty of Medicine, Chulalongkorn University, Bangkok, Thailand; ^8^Division of Nuclear Medicine, Department of Radiology, Faculty of Medicine Siriraj Hospital, Mahidol University, Bangkok, Thailand; ^9^Department of Educational Psychology and Counseling, Faculty of Education, Khon Kaen University, Khon Kaen, Thailand; ^10^Research and Service Institute for Autism, Khon Kaen University, Khon Kaen, Thailand; ^11^Department of Physiology, Faculty of Medicine, Khon Kaen University, Khon Kaen, Thailand

**Keywords:** F-18 FDG PET/CT, brain glucose metabolism, autism spectrum disorders, laterality, treatment by non-invasive brain stimulation, positron emission tomography

## Abstract

**Background and rationale:**

Autism spectrum disorder (ASD) is a neuropsychiatric disorder that has no curative treatment. Little is known about the brain laterality in patients with ASD. F-18 fluorodeoxyglucose positron emission computed tomography (F-18 FDG PET/CT) is a neuroimaging technique that is suitable for ASD owing to its ability to detect whole brain functional abnormalities in a short time and is feasible in ASD patients. The purpose of this study was to evaluate brain laterality using F-18 FDG PET/CT in patients with high-functioning ASD.

**Materials and methods:**

This case-control study recruited eight ASD patients who met the DSM-5 criteria, the recorded data of eight controls matched for age, sex, and handedness were also enrolled. The resting state of brain glucose metabolism in the regions of interest (ROIs) was analyzed using the Q.Brain software. Brain glucose metabolism and laterality index in each ROI of ASD patients were compared with those of the controls. The pattern of brain metabolism was analyzed using visual analysis and is reported in the data description.

**Results:**

The ASD group’s overall brain glucose metabolism was lower than that of the control group in both the left and right hemispheres, with mean differences of 1.54 and 1.21, respectively. We found statistically lower mean glucose metabolism for ASD patients than controls in the left prefrontal lateral (*Z* = 1.96, *p* = 0.049). The left laterality index was found in nine ROIs for ASD and 11 ROIs for the control. The left laterality index in the ASD group was significantly lower than that in the control group in the prefrontal lateral (*Z* = 2.52, *p* = 0.012), precuneus (*Z* = 2.10, *p* = 0.036), and parietal inferior (*Z* = 1.96, *p* = 0.049) regions.

**Conclusion:**

Individuals with ASD have lower brain glucose metabolism than control. In addition, the number of ROIs for left laterality index in the ASD group was lower than control. Left laterality defects may be one of the causes of ASD. This knowledge can be useful in the treatment of ASD by increasing the left-brain metabolism. This trial was registered in the Thai Clinical Trials Registry (TCTR20210705005).

## Introduction

Autism spectrum disorder (ASD) is a neurodevelopmental disorder. There are several aspects listed for the ASD pathology, i.e., the irregular arrangement of neural connections, neuronal migration defects, neurotransmitter imbalance, and altered dendritic morphology (John and Watts, 2008). A systematic review concluded that there is evidence of brain laterality related to ASD (Cara et al., 2022). In addition, a meta-analysis that recruited data from magnetic resonance imaging (MRI), functional MRI (fMRI), diffusion tensor imaging, and magnetoencephalography studies revealed a tentative decrease in the strength of laterality at the inferior frontal gyrus in ASD. However, the results showed an inclusive relationship between ASD and brain laterality (Preslar et al., 2014). Therefore, further studies about hemispheric specialization and brain laterality in ASD are warranted.

F-18 fluorodeoxyglucose positron emission computed tomography (F-18 FDG PET/CT) is another functional neuroimaging technique for evaluating brain metabolism, similar to resting-state fMRI. Previous studies have demonstrated a correlation between these two techniques (Soddu et al., 2016; Savio et al., 2017). However, PET/CT is superior with ASD individuals over resting state fMRI, because PET/CT requires a shorter time period and has no loud radio transmission sounds in the scanner to frighten ASD individuals and make them move. Therefore, PET/CT is more feasible than fMRI for individuals with ASD. In the cellular level, F-18 FDG PET/CT is also a well-established imaging modality for observing glucose metabolism within neuronal cells (Varrone et al., 2009).

Preliminary evidence supports brain laterality in PET scans for individual with normal brain function, which defined as persons without any mental illness or neurological deficit, Willis et al. (2002) reported left laterality in the medial frontal gyrus, posterior thalamus, lingual gyrus, cuneus, and superior cingulate cortex. Right laterality was found in the mesio-anterior cerebellum and lateral frontal and temporal regions normalized by global glucose metabolism during an auditory continuous performance task at 20–69 years old (Willis et al., 2002). Gur et al. (1995) assessed glucose metabolism during a resting state in individual with normal brain function with a mean age of 27.5 years and reported higher metabolism in the left hemisphere for the premotor, motor, and sensorimotor regions and higher right hemispheric glucose metabolism in the cerebellum (Gur et al., 1995). Azari et al. (1992) studied the resting state of brain glucose metabolism in healthy 18–40-year-old subjects using F-18 FDGPET/CT (Azari et al., 1992). They found no differences in the regional or right-left asymmetrical brain glucose metabolism between men and women. These heterogeneous findings resulted from a wide variety of measurement methods, that is, measurements at resting and functioning states, as well as a variety of normalized references.

At present, there are no studies on brain laterality in stocktickerASD patients. Although there was a report showing lower brain glucose metabolism in the medial frontal lobe and cingulate regions, compared to healthy controls (Hazlett et al., 2004). The previous PET scan studies provided basic information about glucose metabolism in the stocktickerASD brain but they did not focus on brain laterality. From the present data, there are some laterality studies on neuropsychiatric disorders (Savic and Lindström, 2008; Morimoto et al., 2013; Pustina et al., 2015). However, to the best of our knowledge, there have been no any reports on brain laterality in individuals with stocktickerASD.

Given this lack of brain laterality information in ASD, we sought to determine a systematic brain laterality in ASD high-functioning individuals to facilitate further understanding of hemispheric specialization in the resting state of the ASD brain.

## Materials and methods

### Study design

This case-control study used F-18FDG PET/CT scans to investigate possible differences in resting-state brain laterality between eight high-functioning ASD individuals and eight matched controls. The study was conducted between July 7, 2020 and October 30, 2021, and all interventions were performed at the Srinagarind Hospital, Faculty of Medicine, Khon Kaen University, Thailand. This trial was registered in the Thai Clinical Trials Registry (TCTR20210705005).

### Participant recruitment and informed consent

The study participants were recruited *via* advertisements at the Institute of Vocational Education Northeastern Region 3 and the Autism Parents Association of Khon Kaen Province, Thailand. The study procedures were described to any eligible participant who expressed an interest in participating in the study by a child psychiatrist (N.P.) according to the DSM-5 clinical assessment using the Autism Diagnostic Observation Schedule-2 (ADOS-2) (Lord et al., 2000).

The inclusion criteria were as follows: (a) mild to moderate autistic symptoms (Childhood Autism Rating Scale, CARS score 30–36.5) where the patient can stay still in the CT scanner during the examination; (b) aged between 15 and 30 years; (c) normal brain structure evaluated by MRI; and (d) the caregiver together with the patients consented to participate in the research.

The exclusion criteria were as follows: (a) severe neurological disorders such as brain tumor, intracranial infection, and uncontrollable epilepsy; (b) uncooperative parents; and (c) claustrophobia.

Prior to starting the study, each participant underwent history taking and physical examination by a neurologist (S.T. or N.A.) to detect comorbid neurological problems. The brain structures of the participants were assessed using MRI, and the ASD functioning class was assessed by a child psychiatrist (N.P.).

The study also included eight recorded images of non-ASD patients who underwent a whole-body F-18 FDG PET/CT scan as a control group (Sharma et al., 2018). All records of non-ASD patients were obtained from the Nuclear Medicine Unit, Khon Kaen University, Thailand. All patients in the control records met the inclusion criteria: (1) age, sex, and handedness matched to the ASD group; (2) use of the same imaging protocol as ASD participants; (3) complete documentation about patients’ history, diagnosis, physical examination, and course of treatments; (4) normal neurologic examinations at the time of the study; (5) normal brain structure evaluated by MRI; and (6) complete image acquisition by PET/CT without significant artifacts. The exclusion criteria were as follows: (1) history of neuropsychiatric diseases such as ASD, depression, schizophrenia, ADHD, mania, and epilepsy; (2) undergoing treatment with neuropsychiatric medications; and (3) CNS impairment, brain structural abnormality, or brain metastasis. Information of the non-ASD patients and their F-18 FDG PET/CT brain images were reviewed and reanalyzed to obtain the uptake ratio in each brain region using Q.Brain software (by K.K.). Verbal consent *via* telephone (N.A.) was obtained from all participants in the control group before data extraction.

The study was conducted in accordance with the Declaration of Helsinki and approved by the Ethics Committee of Khon Kaen University (identifier number: HE 621277). Written informed consent was obtained from all the patients and their caregivers before participation.

## Measures

### Autism spectrum disorder functioning class

The ASD functioning class was classified as having low – or high-functioning autism. High-functioning autism is defined as an individual with ASD who has an intelligence quotient (IQ) above or equal 70. The Wechsler Adult Intelligence Scale fourth edition (WAIS-IV) was used for the IQ test in this study (Alvares et al., 2020).

### PET scan procedure and data processing

The F-18 FDG was produced with a radiochemical purity of > 95%. The imaging equipment used was a PET/CT scanner (Discovery 690; GE Healthcare, WI, United States). Each participant underwent brain F-18 FDG PET/CT imaging after signing an informed consent form and at least 6 h of fasting. All brain F-18 FDG PET/CT images were acquired 45 min after intravenous injection of 7 mCi of F-18 FDG during the resting stage. CT image acquisition parameters were as follows: voltage, 120 kV; tube current, 10 mA; thickness of scanning slice, 3.27 mm; speed, 0.5 sec/rotation; pitch, 0.98; scanning duration, 39.37 s. Third-dimensional PET image acquisition was performed in the same range as the CT scan. The ordered subset expectation maximization method was used for the image reconstruction. The reconstructed data were transferred to a dedicated workstation (GE Xeleris Workstation, version 4.0; GE Healthcare) for both qualitative and quantitative analyses. The 26 cortical regions of interest (ROIs) were automatically located after image fusion and anatomical standardization. The ROIs included the bilateral prefrontal lateral, prefrontal medial, sensorimotor, anterior cingulate, posterior cingulate, precuneus, parietal superior, parietal inferior, occipital lateral, primary visual, temporal lateral, temporal mesial, cerebellum, and pons regions.

Our study measured glucose uptake, which is the amount of the glucose (F-18 FDG) uptake by the neural cells. The glucose metabolism is the amount of glucose utilized by the cells. The neural cells firstly uptake (import) F-18 FDG *via* glucose transporter type 3 (GLUT-3) and then the F-18 FDG is metabolized by glycolysis pathway within the cytoplasm and is stored in the cell. Thus, the glucose (F-18 FDG) uptake directly indicates the brain glucose metabolism (utilization); two terms (glucose (F-18 FDG) metabolism and glucose uptake) can be interchangeable (Varrone et al., 2009).

### Visual analysis

The patterns of brain metabolism were qualitatively assessed after adjusting thalamus and basal ganglia as the maximal intensity regions. Cross-sectional images of brain metabolism were independently reviewed and scored as normal, hypo-, or hypermetabolism by three expert nuclear medicine physicians (D.T., S.T., and B.K.). First, two experts (D.T. and S.T.) independently reviewed and sent the results to K.K., with discordant results sent to another expert (B.K.) who was blinded to the prior reviewers’ results. The evaluation of a third expert was regarded as the final arbitration. The three expert nuclear medicine physicians were also blinded to each other and the clinical status of the participants.

Because of visual analysis was subjective and had no cut-off point, we measured assessor reliability to ensure the quality of the evaluation among the investigator as follows: three nuclear medicine physicians rated the same selected set of 28 brain pet scan images on an assessment document as hyper-, normal-, or hypo-metabolism twice with an inter-assessment interval of 2 months.

### Quantitative analysis

Quantitative analysis of the brain glucose metabolism ratio was calculated for both ASD patients and controls in all predefined ROIs using commercial software with an available database, Q.Brain (GE Healthcare, Milwaukee, WI, United States). The previous studies found many hypometabolic brain areas involving cerebral cortex and cerebellum in patients with ASD (Hampson and Blatt, 2015). Using global metabolism as the reference while many brain areas were in hypometabolic state may cause overestimation of the metabolic value (Yakushev et al., 2008). Therefore, the pons was the most appropriate area to be the reference region (Verger et al., 2021).

### Brain laterality

The 28 cortical ROIs of brain glucose metabolism in both the ASD and control groups were assessed for laterality (by K.K.) using the laterality index [100 × (left-right)/(mean left + mean right)/2] (Willis et al., 2002). Brain laterality is presented as left or right laterality. The laterality index was compared between the ASD and control groups using the Wilcoxon signed-rank test.

### Overall brain glucose metabolism

To obtain an overview of the brain glucose metabolism in each hemisphere, we summed the brain glucose metabolism of all ROIs in each left or right hemisphere. We compared the overall brain glucose metabolism between the ASD and control groups, as well as between the left and right hemispheres of each group using the Wilcoxon signed-rank test.

### Statistical analysis

A previous study reported that the effect size of the mean change difference in regional glucose metabolism was 0.09, with a pooled variance of 0.06 (Hazlett et al., 2004). We calculated that a sample size of eight patients was required to provide a statistical power of 90% to detect the difference between pre – and post-tDCS treatment with a two-sided alpha significance level of 0.05.

Regional brain glucose metabolism by visual analysis is presented as normal, hypo, or hypermetabolism. For other descriptive purposes, the mean and standard deviation of the demographic and outcome variables were calculated. For the validity test, changes in brain metabolism assessed by three independent observers were analyzed using Cronbach’s alpha. The brain glucose metabolism ratio within the control group and ASD group, and the laterality index in each ROI between the ASD and control groups were compared using the Wilcoxon signed-rank test. The correlation between age and brain glucose metabolism (F-18 FDG uptake) across all ROIs has been evaluated using Spearman’s correlation coefficient and the relationship was explored by regression analysis. Regarding to the neuropsychiatric medications that can suppress the neuronal activity and can decrease overall brain glucose metabolism, the second analysis between ASD with medications and ASD without medications was performed using Wilcoxon signed-rank test. Analyses were conducted using SPSS Statistics (SPSS version 20), with the significance level set at *p* < 0.05. The data were monitored by P.A. and K.K.

## Results

### Demographic data

The eight ASD participants had a mean (± SD) age of 19.00 (± 3.02) years (range, 15–23) and a mean age of 4.87 (± 3.89) years at ASD diagnosis (range, 1.5–11). IQ means (± SD) is 86.45 (± 10.49) (range, 73–105). Most of the participants were male (87.5%) and left-handed (63%); one had an ASD family history (12.5%), one participant had a perinatal risk factor (12.5%), and six participants had idiopathic or no risk factors detected (75%). Four participants (50%) were born *via* cesarean section. There was nobody suffering from any psychiatric disorders. However, there was one participant who had focal epilepsy as the underlying disease. She was ensured by neurologists (S.T. and N.A.) that her status was controllable because she received only one antiepileptic drug (Lacosamide) and did not have any episode of epileptic attack for more than 1 year (Kwan et al., 2010). The mean blood glucose levels before performing F-18 FDG PET-CT were 96.75 ± 11.49 mg/dL.

The control group had a mean (± SD) age of 19.00 (± 2.83) years (range, 16 to 23). The participants were diagnosed with T-cell lymphoma (37.5%), Hodgkin lymphoma (12.5%), and non-Hodgkin lymphoma (50%), all of whom were in stage I with complete remission and were at the 1-year follow-up. None of the participants in the control group had neurological symptoms, and all participants (both the control and ASD groups) had normal brain structures as evaluated by MRI. The mean blood glucose levels before performing F-18 FDG PET-CT were 94.82 ± 15.31 mg/dL. The demographic data of the participants are presented in Table 1.

**TABLE 1:** Demographic data of participants.

Autism spectrum disorders (*n* = 8)												Control (*n* = 8)>						
ID	Sex	Age (years)	Handed ness	Age of diagn osis	Parturi tion	Maternal age at parturi tion	Paternal age at parturi tion	History of preg nancy	Family history for ASD	Treatment		ID	Sex	Age (years)	Handed ness	Diag nosis	Treatment	

										Medica tion	Behavioral therapy						Chemo therapy	Status
**ASD 01**	F	19	R	3.5	C/S	33	40	Normal	Yes	Ri, B, L	SB	Ctrl 01	F	17	R	N-HL	Complete	Remission
**ASD 02**	M	15	L	11	C/S	28	38	PE	No	Ri	SB	**Ctrl 02**	M	16	L	TCL	Complete	Remission
ASD 03	M	16	R	3.25	N/L	34	34	Normal	No	No	SB	Ctrl 03	M	17	R	ALL	Complete	Remission
**ASD 04**	M	22	R	2.5	C/S	34	32	Normal	No	No	OT	**Ctrl 04**	M	22	R	N-HL	Complete	Remission
ASD 05	M	18	L	4.25	N/L	26	30	Normal	No	No	SB	Ctrl 05	M	18	L	HL	Complete	Remission
**ASD 06**	M	22	L	1.92	N/L	30	35	Normal	No	No	OT	**Ctrl 06**	M	22	L	N-HL	Complete	Remission
ASD 07	M	17	L	1.5	C/S	40	50	Normal	No	No	OT	Ctrl 07	M	17	L	N-HL	Complete	Remission
**ASD 08**	M	23	L	11	N/L	33	33	Normal	No	Ri	No	**Ctrl 08**	M	23	L	TCL	Complete	Remission

ASD, autism spectrum disorders; B, Benzhexol; C/S, cesarean section; Ctrl, control; F, female; HL, Hodgkin lymphoma; L, lacosamide; L, left; M, male; N-HL, non-Hodgkin lymphoma; N/L, normal labor; OT, occupational therapy; PE, pre-eclampsia; R, right; Ri, risperidone; SB, school base; TCL, T-cell lymphoma.

### Brain glucose metabolism assessed by visual analysis

**FIGURE 1:**
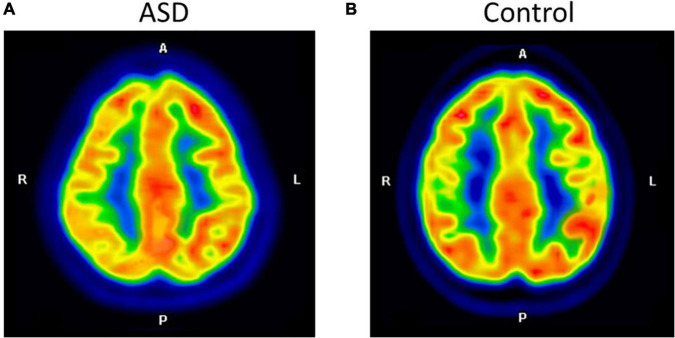
The axial images of the F-18 FDG PET/CT scan demonstrated relatively decreased FDG uptake at the right parietal inferior compared to the left parietal inferior in ASD **(A)**. The control participant shows normal FDG uptake in the same region **(B)**.

Prior to evaluating regional brain glucose metabolism, we assessed the inter-observer and intra-rater reliabilities for visual analysis. There was a very high degree of reliability among the three investigators (*r* = 0.87, 95%CI = 0.76 to 0.94, *p* < 0.001). The intra-rater reliability test was also high for D.T. (*r* = 0.86, 95%CI = 0.69 to 0.93, *p* < 0.001), S.T. (*r* = 0.94, 95%CI = 0.88 to 0.97, *p* < 0.001), and B.K. (*r* = 1.00, 95%CI = 1.00 to 0.98, *p* < 0.001). There were several hypometabolic areas, particularly in the right cerebral hemisphere (8/8), in the ROIs examined by visual analysis in all eight ASD participants (Figure 1 and Table 2). Normal brain glucose metabolism was reported by reviewers in all ROIs of the control group.

**TABLE 2:** Visual analysis of autism spectrum disorders’ PET/CT image (*n* = 8).

ID	Area of hypometabolism	Area of hypermetabolism
ASD 01	R – Temporal lateral	Bi- Visual cortex
ASD 02	R – Parietal superior	Bi- Visual cortex
ASD 03	R – Parietal superior,	Bi- Visual cortex
	R – Temporal lateral	
ASD 04	R – Parietal superior,	Bi- Visual cortex
	R – Parietal inferior,	
	R – Temporal lateral	
ASD 05	Bi – Posterior cingulate cortex,	Bi- Visual cortex
	Bi – Precuneus,	
	Bi – Parietal superior,	
	Bi – Parietal inferior	
ASD 06	R – Parietal superior,	Bi- Visual cortex
	R – Parietal inferior	
ASD 07	R – Parietal superior,	Bi- Visual cortex
	R – Temporal lateral	
ASD 08	R – Parietal superior,	Bi- Visual cortex
	R – Temporal lateral	

ASD, autism spectrum disorders; Bi, bilateral; L, left; R, right.

### Brain glucose metabolism comparing between autism spectrum disorder and control

Autism spectrum disorder overall brain glucose metabolism was lower than that of controls in both the left and right hemispheres, with mean differences of 1.54 and 1.21, respectively. For the left ROIs, the Wilcoxon signed-rank test showed statistically lower mean glucose metabolism for ASD patients than controls in the left prefrontal lateral (*Z* = 1.96, *p* = 0.049) and trends toward a lower mean glucose metabolism in the left anterior cingulate (*Z* = 1.68, *p* = 0.092), left posterior cingulate (*Z* = 1.68, *p* = 0.093), left parietal superior (*Z* = 1.68, *p* = 0.093), left parietal inferior (*Z* = 1.82, *p* = 0.069), and left temporal mesial area (*Z* = 1.68, *p* = 0.092).

**FIGURE 2:**
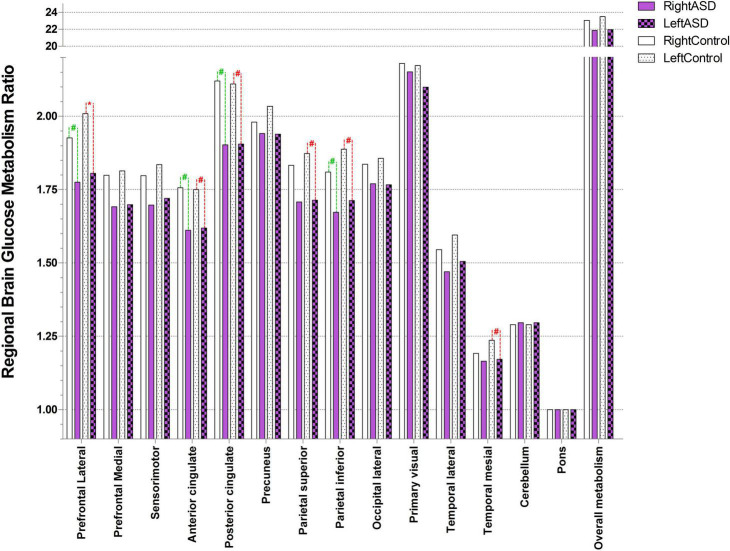
Ratio of brain glucose metabolism in regions of interest between control and ASD (* represents statistically significant, # tentative statistically significant by Wilcoxon signed-rank test).

For the right ROIs, there was no statistical difference in the mean glucose metabolism between the ASD and control groups. However, there was a trend of lower brain glucose metabolism for ASD than control in the right prefrontal lateral (*Z* = 1.82, *p* = 0.069), right anterior cingulate (*Z* = 1.68, *p* = 0.093), right posterior cingulate (*Z* = 1.68, *p* = 0.093), and right parietal inferior area (*Z* = 1.69, *p* = 0.091) (Figure 2 and Table 3).

**TABLE 3:** Brain glucose metabolism in the right and left region of interest presented as ratio of brain glucose metabolism using Q.Brain software in ASD (*n* = 8) and control (*n* = 8).

	Prefrontal lateral		Prefrontal medial		Sensorimotor		Anterior cingulate		Posterior cingulate		Precuneus		Parietal superior		Parietal inferior		Occipital lateral		Primary visual		Temporal lateral		Temporal mesial		Cerebellum	Pons	Overall metabolism	
															
	R	L	R	L	R	L	R	L	R	L	R	L	R	L	R	L	R	L	R	L	R	L	R	L	M	M	R	L
**ASD 01**	1.5	1.54	1.43	1.44	1.48	1.52	1.31	1.3	1.69	1.7	1.76	1.73	1.51	1.5	1.47	1.5	1.6	1.61	1.96	1.9	1.28	1.32	1.01	1.03	1.2	1	19.2	19.29
**ASD 02**	1.88	1.91	1.8	1.79	1.74	1.76	1.6	1.64	2.08	2	2.03	2	1.84	1.9	1.83	1.85	1.87	1.84	2.04	1.86	1.64	1.65	1.28	1.2	1.28	1	22.91	22.68
ASD 03	1.96	1.96	1.8	1.82	1.84	1.84	1.71	1.74	1.88	1.89	1.88	1.89	1.61	1.62	1.77	1.79	1.84	1.83	2.3	2.15	1.57	1.6	1.27	1.28	1.4	1	22.83	22.81
**ASD 04**	1.74	1.72	1.67	1.67	1.7	1.69	1.77	1.78	1.72	1.76	1.89	1.78	1.69	1.62	1.6	1.63	1.73	1.73	2.26	2.14	1.45	1.47	1.13	1.18	1.36	1	21.71	21.53
ASD 05	1.69	1.72	1.55	1.56	1.58	1.63	1.52	1.54	1.84	1.8	1.78	1.79	1.58	1.65	1.54	1.56	1.69	1.71	1.94	2.08	1.38	1.4	1.13	1.16	1.25	1	20.47	20.85
**ASD 06**	1.64	1.73	1.66	1.63	1.6	1.61	1.48	1.45	1.85	1.91	1.96	2.01	1.71	1.67	1.6	1.67	1.68	1.67	2.16	2.1	1.35	1.42	1.11	1.13	1.26	1	21.06	21.26
ASD 07	1.8	1.84	1.72	1.74	1.71	1.81	1.71	1.71	1.92	1.95	1.96	1.97	1.73	1.76	1.72	1.75	1.81	1.79	2.15	2.2	1.47	1.52	1.11	1.14	1.25	1	22.06	22.43
**ASD 08**	1.99	2.02	1.9	1.94	1.93	1.9	1.79	1.79	2.24	2.23	2.27	2.28	1.99	1.99	1.85	1.95	1.94	1.95	2.4	2.36	1.62	1.66	1.28	1.25	1.37	1	24.57	24.69
Mean	1.78	1.81	1.69	1.70	1.70	1.72	1.61	1.62	1.90	1.91	1.94	1.93	1.71	1.71	1.67	1.71	1.77	1.77	2.15	2.10	1.47	1.51	1.17	1.17	1.30	1.00	21.85	21.94
**SD**	0.18	0.18	0.17	0.18	0.16	0.16	0.2	0.17	0.17	0.19	0.18	0.18	0.18	0.17	0.15	0.19	0.14	0.15	0.2	0.18	0.15	0.14	0.09	0.1	0.07	0	1.65	1.60
Control 01	2.02	2.09	1.93	1.93	1.95	1.99	1.75	1.80	2.15	2.15	2.11	2.17	1.94	1.99	1.9	1.94	2.02	2.01	2.27	2.29	1.64	1.70	1.25	1.30	1.33	1.00	24.26	24.69
**Control 02**	1.96	2.03	1.91	1.95	1.89	2.00	1.85	1.87	2.26	2.26	2.08	2.12	1.97	2.02	1.9	1.93	1.99	2.00	2.43	2.43	1.61	1.63	1.18	1.23	1.39	1.00	24.42	24.86
Control 03	2.30	2.35	2.02	2.02	1.99	2.02	2.08	1.99	2.50	2.58	2.04	2.25	1.92	1.97	2.05	2.17	1.81	1.88	2.17	2.03	1.66	1.67	1.30	1.30	1.19	1.00	25.03	25.42
**Control 04**	1.84	1.94	1.71	1.71	1.66	1.76	1.62	1.66	2.04	2.01	1.93	1.93	1.76	1.77	1.74	1.87	1.68	1.66	1.83	1.87	1.47	1.51	1.18	1.22	1.30	1.00	21.76	22.21
Control 05	1.78	1.83	1.75	1.75	1.72	1.69	1.76	1.77	2.06	1.99	1.94	1.96	1.75	1.82	1.7	1.74	1.77	1.78	2.19	2.13	1.46	1.50	1.14	1.18	1.31	1.00	22.33	22.45
**Control 06**	1.94	2.11	1.75	1.77	1.78	1.86	1.70	1.72	2.12	2.08	2.11	2.16	1.96	1.97	1.86	1.97	1.94	1.98	2.24	2.39	1.59	1.66	1.21	1.29	1.25	1.00	23.45	24.21
Control 07	1.82	1.89	1.62	1.66	1.71	1.73	1.60	1.65	1.96	2.05	1.88	1.89	1.68	1.70	1.72	1.79	1.69	1.73	2.01	2.01	1.48	1.54	1.15	1.23	1.26	1.00	21.58	22.13
**Control 08**	1.75	1.83	1.70	1.72	1.68	1.63	1.63	1.60	1.87	1.86	1.75	1.79	1.68	1.74	1.61	1.69	1.79	1.81	2.30	2.23	1.45	1.55	1.12	1.14	1.29	1.00	21.62	21.88
Mean	1.93	2.01	1.80	1.81	1.80	1.84	1.75	1.76	2.12	2.12	1.98	2.03	1.83	1.87	1.81	1.89	1.84	1.86	2.18	2.17	1.55	1.60	1.19	1.24	1.29	1.00	23.06	23.48
**SD**	0.18	0.18	0.14	0.13	0.13	0.15	0.16	0.13	0.19	0.22	0.13	0.16	0.13	0.13	0.14	0.15	0.13	0.13	0.19	0.20	0.09	0.08	0.06	0.06	0.06	0.00	1.40	1.45
C-ASD	0.151#	0.20*	0.11	0.12	0.10	0.12	0.14#	0.14#	0.22#	0.22#	0.04	0.10	0.13	0.159#	0.14#	0.18#	0.07	0.09	0.03	0.07	0.07	0.09	0.03	0.07#	−0.01	0.00	1.21	1.54

ASD, autism spectrum disorders group; C – ASD, mean of control group minus mean of ASD group; L, left; M, middle part; N/A, not applicable; R, right. * Represents statistical difference p < 0.05; # Represent tentative statistical difference 0.09 > p > 0.05 by Wilcoxon signed-rank test. The bold values are the mean of brain glucose metabolism.

#### Overall brain glucose metabolism between autism spectrum disorder with and without medications

Wilcoxon signed-rank test of the overall brain glucose metabolism showed no statistical difference between two groups (*Z* = 0.54, *p* = 0.593).

### Brain laterality index

**FIGURE 3:**
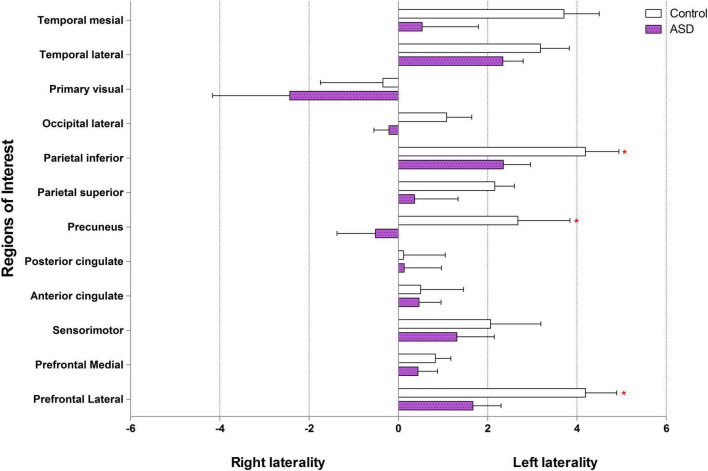
Brain laterality in regions of interest between control and ASD (* represents statistically significant by Wilcoxon signed-rank test).

In the control group, our data showed left laterality index in 11 ROIs, that is, prefrontal lateral, prefrontal medial, sensorimotor, anterior cingulate, posterior cingulate, precuneus, parietal superior, parietal inferior, occipital lateral, temporal lateral, and temporal mesial. However, in the ASD group, the left laterality index was found in only nine ROIs: the prefrontal lateral, prefrontal medial, sensorimotor, anterior cingulate, posterior cingulate, parietal superior, parietal inferior, temporal lateral, and temporal mesial. Wilcoxon signed-rank test showed a significantly lower degree of left laterality for ASD over controls in the prefrontal lateral (*Z* = 2.52, *p* = 0.012), precuneus (*Z* = 2.10, *p* = 0.036), and parietal inferior (*Z* = 1.96, *p* = 0.049). There was a trend toward a lower degree of left laterality in the parietal superior (*Z* = 1.68, *p* = 0.093) and temporal mesial regions in the ASD group than in the control group (*Z* = 1.82, *p* = 0.069). Brain laterality in the region of interest between the control and ASD groups is shown in Figure 3.

### The correlation between age and brain glucose metabolism

In the control group, Spearman’s correlation coefficient showed a trend toward association between age and brain glucose metabolism at the left prefrontal medial (*p* = 0.071, *r* = 0.667) and right temporal lateral (*p* = 0.072, *r* = 0.665). No association between age and brain glucose metabolism in any ROI of ASD group.

## Discussion

To the best of our knowledge, this is the first study that aimed to study brain laterality using F-18 FDG PET/CT normalized by pons in high-functioning ASD individuals compared to controls (normal neurocognitive subjects matched for age, sex, and handedness). This study revealed that brain glucose metabolism at the left prefrontal lateral in the ASD group was lower than the control group. We also found a statistically significant decrease in left laterality in ASD patients compared to controls at the prefrontal lateral, precuneus, and parietal inferior regions.

### Why brain glucose metabolism in autism spectrum disorder is lower than control?

Our visual analysis found hypometabolic areas (lower brain glucose metabolism or reduced glucose metabolism) at the right ROIs in all ASD participants than in controls. ASD pathology is the impairment of synaptogenesis (Courchesne et al., 2020), synaptic pruning (Tang et al., 2014; Kim et al., 2016), and alterations in neural network proliferation (Marchetto et al., 2016). The study using resting-state fMRI evidenced both hypo and hyper-connectivity in ASD. These findings indicated that one of the causes of ASD is abnormal neural connections (Haghighat et al., 2021). F-18 FDG PET/CT scan is one of the studies for detection of abnormal brain glucose metabolism. Neuronal cells that perform more action always have higher glucose metabolism, as shown in the PET images. In contrast, less active or non-functioning cells were identified as hypometabolic areas. Our observation revealed multiple areas with lower brain glucose metabolism, including the parietal superior, temporal lateral, parietal inferior, and precuneus regions in the ASD. These findings support those of previous studies, including Hazlett et al. (2004), who reported cortical lower glucose metabolism in the medial prefrontal and anterior cingulate regions in ASD patients compared with controls (Hazlett et al., 2004). Another study showed lower brain glucose metabolism in the amygdala, hippocampus, parahippocampal gyrus, caudate nucleus, cerebellum, mesial temporal, thalamus, superior temporal, and mesial temporal regions (Sharma et al., 2018). A lower brain metabolism had also been reported in the hippocampus, amygdala, parahippocampal gyrus, mesial temporal, parietal, cerebellum (Leblanc, 2017) parietal lobe, temporal lobe, anterior cingulate, posterior cingulate, and thalamus (Haznedar et al., 2006; Manglunia and Puranik, 2016; Mitelman et al., 2018). Our study could not focus on the hippocampus and amygdala due to limitation of the processing software. As we have already known, these two brain regions are very important for ASD pathology. As mentioned in the previous study, the volume of hippocampus combined with amygdala are approximately 87% of all element in mesial temporal region (Striepens et al., 2010). Therefore, decrease in both brain glucose metabolism and brain laterality at the left temporal mesial in our ASD participants was sufficient to infer that there was abnormal brain glucose metabolism at the hippocampus and amygdala. Nevertheless, using the specific software that can analyze the hippocampus and amygdala separately would be more accurate.

We also observed areas with higher brain glucose metabolism in both primary visual cortices. These finding may associate with local functional disruption within the occipital cortices, which could produce spontaneous brain activity or spontaneous ability to perform a given cognitive or physiological tasks, causing abnormally high brain glucose metabolism in these regions (Itahashi et al., 2015). Overall, the lower brain glucose metabolism in the ASD group compared to the control group in our study was probably due to abnormal neuronal connectivity (Billeci et al., 2013; Haghighat et al., 2021).

### What is the pathophysiology of brain laterality?

Our data in the control (individual with normal brain function) group showed left laterality in almost all ROIs except primary visual area using Q.Brain software normalized by pons. The prefrontal lateral, prefrontal medial, sensorimotor, anterior cingulate, posterior cingulate, precuneus, parietal superior, parietal inferior, occipital lateral, temporal lateral, and temporal mesial showed left laterality in various degree by which the prefrontal lateral was the most left lateralization.

At present, there are some studies that evaluate laterality in individual with normal brain function. They usually presented the laterality using three aspects (1) hemispheric lateralization; (2) functional lateralization, i.e., language, sensory, and motor lateralization; and (3) ROI lateralization. The hemispheric and functional lateralization are extensively used and usually measured by fMRI. The ROI lateralization, as used in our study, is rarely mentioned but it can be measured by PET scan (Gur et al., 1995; Willis et al., 2002). Among the ROI lateralization by PET scan studies, there was also a difference in reference region used in the study. Gur RC and colleagues demonstrated left laterality in almost all ROI including the premotor cortex, sensorimotor, and parietal superior in their study using global as a reference region. They also reported right laterality in the occipital lateral (Gur et al., 1995). Willis MW and colleagues, using global reference, demonstrated left laterality in the frontal medial and precuneus regions. However, they found right laterality in the frontal lateral and temporal regions (Willis et al., 2002). According to our study that used pons as a reference region, it was difficult to compare our results with these two studies. We therefore perform second analysis using global as reference region (Table 4 in supplemental document) in order to compare the results. The results of our study were not different from our results that used pons as a reference region. Comparing with Gur’s and Willis’ studied, the left laterality was found in most of the ROIs in the individual with normal brain function. However, right laterality was found in different regions for example; Willis found right laterality in the frontal lateral, while our study and Gur evidenced left laterality in frontal lateral. This difference may be due to (1) Willis performed in volunteers with mean age of 40 years old, while Gur and our studies performed in the individual with normal brain function with mean age about 20 years old. Changes in glucose metabolism due to age-related differences could be affected frontal lateral region (Fujimoto et al., 2008; Hsieh et al., 2012); and (2) the differences in protocols; our study and Gur study measured brain glucose metabolism at the resting stage, while Willis’ study measured during tasks assignment. Although studies in this area do not have enough evidences to draw a conclusion, individuals with normal brain function are likely to have left laterality in most ROIs.

**TABLE 4:** Brain laterality in control group using global as the reference region (*n* = 8).

	Prefrontal Lateral	Prefrontal medial	Sensori motor	Anterior cingulate	Posterior cingulate	Precu neus	Parietal superior	Parietal inferior	Occipital lateral	Primary visual	Temporal lateral	Temporal mesial
Control 01	3.75	0.00	2.03	2.10	0.00	2.71	2.95	2.00	0.00	0.86	3.52	4.57
Control 02	3.75	2.05	5.08	1.05	0.00	1.80	1.97	2.00	1.00	0.00	2.35	4.57
Control 03	2.81	0.00	2.03	−4.20	−0.86	−1.31	−1.95	2.99	4.01	−6.03	1.17	0.00
Control 04	3.75	0.00	6.09	2.10	–1.73	0.90	0.00	3.99	–1.00	1.72	2.35	3.05
Control 05	2.81	1.02	−1.02	0.00	−0.86	1.80	1.97	2.00	2.00	−0.86	3.52	3.05
Control 06	2.81	0.00	4.06	1.05	–1.73	–2.71	–2.92	4.99	2.00	6.90	4.70	0.00
Control 07	0.94	2.05	1.02	0.00	5.19	0.00	0.98	2.99	2.00	−0.86	3.52	7.62
Control 08	1.88	–2.05	–3.05	0.00	0.00	0.00	–1.97	3.99	1.00	–3.45	2.35	4.57
Mean	2.81	0.38	2.03	0.26	0.00	0.40	0.13	3.12	1.38	−0.22	2.94	3.43
SD	1.00	1.33	3.07	2.01	2.22	1.79	2.19	1.12	1.51	3.79	1.09	2.54

The negative values represent right laterality and the positive values represent left laterality.

### Why is the left laterality usually found in the resting state of individual with normal brain function?

The lateralized circuits arise from the gene that regulates neural circuit formation but the core concept of genetic mechanism in brain laterality is still unknown (Francks, 2015). The primate mirror system responds to intentional movement in daily activity and imitation of another individual action (Rizzolatti and Sinigaglia, 2010). Moreover, the language circuit relates to the idea of language perception in both verbal and non-verbal communications (Corballis, 2017a). All of these features, located within the left cerebral hemisphere, have been developing over time since the beginning of humankind causing left lateralization (Corballis, 2017a,b).

### Brain laterality in autism spectrum disorder vs. individual with normal brain function?

In the control group, our findings showed left laterality that was observed in all ROIs (except the primary visual region). The left laterality in ASD was found to be lower than that in the control group in the prefrontal lateral, precuneus, and parietal inferior. In addition, we found a trend toward association between age and brain glucose metabolism at the left prefrontal medial and right temporal lateral in the control group while there was no correlation between age and F-18 FDG PET/CT uptake in ASD group.

Brain glucose metabolism in ASD patients were lower than those in controls, as mentioned above. As there have been no previous studies on laterality by FDG PET/CT in individuals with ASD, it was impossible to compare results with the same neuroimaging method. fMRI is another neuroimaging modality similar to PET/CT, and for the ASD preliminary results, we found a decrease in the left laterality index in the prefrontal lateral, precuneus, and parietal inferior regions compared to the control. Our findings are in concordance with other evidence of less laterality in the frontal language areas detected by fMRI (Preslar et al., 2014). The explanation of abnormal left laterality occurring within the ASD brain is unclear, but there is a possibility that atypical early brain development occurred in those brain regions (Benkarim et al., 2021), resulting in abnormal functional lateralization for language and social function in ASD patients (Lindell and Hudry, 2013). As our findings showed decreased brain glucose metabolism of left prefrontal lateral in ASD. We also found decreasing of left laterality in prefrontal lateral. Therefore, this part of the brain is probably important for ASD pathophysiology. As we know that the dorsolateral prefrontal cortex plays an important role in executive function by connecting the frontal and parietal cortices *via* frontoparietal networks (Haber et al., 2021), enhancing working memory capacity (Osaka et al., 2021), and is related to human intelligence (Hearne et al., 2016). An irregular arrangement of neural connections is one of the proposed pathologies of ASD. This abnormal connection may occur mainly in the frontoparietal network and result in decreased brain glucose metabolism in both cerebral hemispheres, but more striking in the left hemisphere, leading to decreased left laterality in the left precuneus and parietal inferior regions, as seen in our ASD participants.

Studies on laterality in individual with normal brain function show heterogeneity, and the results are inconclusive. Our study showed that ASD patients had a distinctive laterality trend compared to controls, that is, a lesser number of ROIs for left laterality. Moreover, among the areas of left laterality found in this study, ASD individuals showed lower brain glucose metabolism levels than control, that is, left laterality index in the prefrontal lateral in control was equal to 4 vs. ASD equal to 1.8. This finding is likely caused by abnormal brain development that results in abnormal left laterality through various mechanisms during the perinatal period (Wiper, 2017).

### How is this new knowledge useful?

Currently, there is no curative treatment for ASDs. Non-invasive brain stimulation (NIBS) is an emerging tool for the treatment of ASDs. The main NIBS devices for ASD treatment are transcranial magnetic stimulation (TMS) and transcranial direct current stimulation (tDCS). A recent systematic review and meta-analysis suggested that TMS might be a novel technique which has therapeutic potential to correct the abnormal neuroplasticity in ASD (Huashuang et al., 2022). Same as TMS, systematic review and meta-analytic study of tDCS identified improvements in social, health, and behavioral problem domains of the Autism Treatment Evaluation Checklist (García-González et al., 2021).

Transcranial magnetic stimulation is neurostimulation using a magnetic field transmitted through the scalp, and induced an electric field to the neuronal cells. The magnitude of this electric field is enough to produce depolarization of neurons. Repetitive TMS (rTMS) delivers multiple series of short magnetic pulse over a specified brain region. The low frequency rTMS leads to long-term suppression of brain excitability, whereas at high frequency rTMS typically results in long-term facilitation of brain excitability by mechanisms associated with long-term potentiation (Khaleghi et al., 2020).

These after effects, which are mediated by glutamatergic synapses [especially the N-methyl-D-aspartate (NMDA) receptors], have been related to long-term potentiation and long-term depression-liked mechanisms. The tDCS is a neuromodulation method that modify neuronal membrane potential and thus affect spontaneous firing rates by applying a weak direct current *via* two electrodes. The anodal electrode will increase excitability of the underneath neurons; whereas, cathodal electrode will decrease excitability of the neuron cell due to hyperpolarization. The effects of tDCS are not only restricted to the area under the electrode but also transmitted by functional connection within the brain to distance areas. The neuroplasticity induced by tDCS might be associated with the increased functional connectivity in the brain (García-González et al., 2021). The primary treatment principle for both devices is to stimulate the less-active brain regions and inhibit the overactive brain regions. The currently targeted brain regions for the NIBS method in ASD patients remain diverse (Barahona-Corrêa et al., 2018). In general, positive clinical outcomes are associated with anodal (stimulation electrode) over the left DLPFC (Schneider and Hopp, 2011; Amatachaya et al., 2014, 2015; Gómez et al., 2017; Auvichayapat et al., 2020; Hadoush et al., 2020). However, another study also showed a positive effect of the cathodal (inhibitory electrode) over the left DLPFC (Rothärmel et al., 2019). To date, studies have been inconsistent regarding the appropriate regional protocol for NIBS electrode placement.

Our study suggests that ASD participants have lower regional brain glucose metabolism in many areas of the brain, as well as less left laterality, than controls. To induce normal brain metabolism, the application of anodal tDCS or high-frequency TMS over the left hemisphere of ASD patients might be more effective than over the right hemisphere or performing left-sided inhibition. Applying these knowledges may extend to the treatment of major depressive disorder, bipolar depression (Cotovio et al., 2022), schizophrenia (Oertel et al., 2010), and other laterality abnormality (He et al., 2022). However, further in-depth studies, including clinical outcomes after non-invasive brain stimulation treatment should be evaluated by functional neuromolecular imaging study in the specific ROI such as MRS, are recommended.

### Limitation of the study

This study could not include normal young-adult participants as the control due to the ethical concerning about radiation exposure. Although patients with lymphoma were found to have global brain hypometabolism, the previous study revealed that these hypometabolic states recovered after sessions of chemotherapy (Nonokuma et al., 2014). In addition, our control participants had completed a full course of chemotherapy before the time of the study and all of them were in the complete remission state. Therefore, in our opinion, it was acceptable to be the control group.

We did not find the difference of overall brain glucose metabolism between the medication and non-medication groups. However, the neuropsychological medications affect the neuronal activity by suppression of overall brain glucose metabolism. In order to precisely answer the research question, our suggestion is to exclude ASD with medications in the future functional neuroimaging study.

### Summary

This study provides preliminary evidence showing lower resting brain glucose metabolism in ASD patients and less left laterality than controls when measured by F-18 FDG PET/CT. In addition, the degree of left laterality in ASD patients was also lower than that in controls. This preliminary knowledge could be usefully applied in ASD treatment by increasing left-brain metabolism and promoting more left laterality. Nonetheless, a larger sample size and clinical outcome-focused studies in combination with neuroimaging in terms of brain laterality are strongly suggested.

## Data availability statement

The raw data supporting the conclusions of this article will be made available by the authors, without undue reservation.

## Ethics statement

The studies involving human participants were reviewed and approved by the Ethics Committee of Khon Kaen University (Identifier number: HE 621277). Written informed consent to participate in this study was provided by the participants’ legal guardian/next of kin.

## Author contributions

PA, DT, NA, and KK: study design. PA, KK, NP, CS, NA, and STi: subject enrollment. CS, NP, KK, DT, STe, and BK: outcome evaluator. PA, KK, and YR: data collection. PA and KK: data analysis. KK, PA, NP, NA, and SP: manuscript writing. All authors contributed to the article and approved the submitted version.
